# Ovarian follicular and luteal dynamics during natural and hormonally induced estrus in *Arunachali yak* (*Bos grunniens*)

**DOI:** 10.3389/fvets.2026.1967091

**Published:** 2026-10-12

**Authors:** Mokhtar Hussain, Lalit Kumar Patel, Arunoday Das, Supriya Bera, Dhrubajyoti Borpujari, Pranjal Borah, Dinamani Medhi, Vijay Paul, Mihir Sarkar

**Affiliations:** 1 ICAR-National Research Centre on Yak, Dirang, Arunachal Pradesh, India; 2 College of Veterinary Science, AVFU, Guwahati, Assam, India; 3 Lakhimpur College of Veterinary Science, AVFU, Joyhing, Assam, India

**Keywords:** *Arunachali yak*, estrous cycle, follicular dynamics, luteal dynamics, ultrasonography

## Abstract

This study investigated follicular growth patterns, corpus luteum dynamics and endocrine profiles during natural and hormonally induced estrous cycles in *Arunachali yak* maintained at the ICAR–NRC on Yak, Nyukmadung, Arunachal Pradesh, India. Thirty-six apparently healthy, cyclic female yaks of second to fourth parity were equally assigned to natural estrus (Group I) or hormonally induced estrus using a modified Ovsynch protocol (Group II). Daily transrectal ultrasonography was performed throughout one estrous cycle to characterize follicular waves and luteal dynamics, along with serial endocrine profiling. Three follicular-wave patterns were observed in natural cycles and four in induced cycles. A two-wave pattern predominated in natural estrus (66.67%), whereas a three-wave pattern predominated following hormonal induction (77.78%). The mean estrous cycle was significantly longer in induced than natural cycles (22.50 ± 0.35 vs. 20.00 ± 0.70 days). The first follicular wave was the longest in both groups, with successive waves showing progressively shorter durations. Corpus luteum size differed significantly between groups, indicating altered luteal dynamics following hormonal induction. Endocrine profiling further showed higher progesterone (5.75 ± 0.13) during induced cycles, increased estradiol (29.20 ± 3.19) at subsequent estrus and the third follicular peak, increased IGF-1 (124.63 ± 2.97) during the mid-and late-luteal phases and elevated cortisol (3.47 ± 0.25) during the early-to-mid luteal phase. Overall, hormonal induction altered follicular-wave patterns, cycle length, luteal development and endocrine dynamics in *Arunachali yak*. These findings facilitate the optimization of estrus synchronization and fixed-time artificial insemination (FTAI) protocols, thereby enhancing ovulatory precision and overall reproductive performance in yaks (*Bos grunniens*).

## 1 Introduction

Yak are seasonal polyestrous animals whose mating season in their native high-altitude environment typically occurs between July and November (1). Their estrous cycle (19–21 days) comprises four phases, viz*.,* proestrus (1–5 days), estrus (12–36 h), metestrus, and diestrus, with ovulation occurring approximately 12–24 h after the end of estrus (2). Yaks have an average inter-calving interval of about 2 years (3). However, recent studies have highlighted the unique reproductive physiology of yak and the challenges associated with improving reproductive efficiency under high-altitude conditions (4). For instance, yak primarily depend on natural grazing resources, and therefore seasonal variation in pasture availability significantly influences their nutritional status and reproductive performance (5, 6). Therefore, improvement of reproductive efficiency in yak is essential for enhancing both the livelihood security of high-altitude communities and the economic sustainability of yak production systems. Yaks suffer from many reproductive problems such as repeat breeding, postpartum anestrus, poor estrus expression, and delayed sexual maturity (1). In addition, a prolonged calving-to-estrus interval is commonly reported and further contributes to reduced reproductive performance (7). Behavioral signs of estrus in yak are also often subtle and short-lived, usually lasting only a few hours during the estrous cycle. These reproductive limitations significantly affect herd productivity and remain a major constraint in yak production systems (4). In fact, under natural breeding conditions, yak exhibit a relatively low annual fertility rate of approximately 40–60% (8).

Artificial insemination is an effective tool that can be utilized in yak for genetic improvement and enhancing productive traits within a short period (3). However, the success rate of artificial insemination (AI) in *Arunachali yak* remains limited due to poor estrus expression, the unpredictable timing of ovulation, and difficulty in estrus detection (3, 9). Therefore, the development of estrus synchronization protocols that enable fixed-time artificial insemination (FTAI) is important for improving reproductive efficiency in yak populations (8), and, over the past few decades, several protocols have been developed for this purpose. Similar reproductive challenges have been reported in buffaloes, where estrus is often weakly expressed, difficult to detect and variable in duration, making prediction of the time of ovulation and determination of the appropriate timing for artificial insemination is challenging. In buffaloes, follicular development and the presence of a dominant follicle has been evaluated by ultrasonography before FTAI to identify females with a higher probability of ovulation and increase the efficiency of timed insemination. These observations stress the importance of understanding follicular dynamics and ovulation timing in yak, where clinical signs of estrus may be poorly expressed (10).

The introduction of ultrasonography in the late 1980s allowed the detailed study of ovarian follicular dynamics (11), which describes the continuous process of follicular growth and regression (12). During this process, primordial follicles develop sequentially into primary, secondary, and eventually tertiary (antral) follicles (13). Although follicular growth patterns have been extensively studied in cattle using ultrasonography, information on ovarian follicular dynamics in yak is still limited, particularly under high-altitude conditions (14).

The concept of follicular wave theory suggests that a group of follicles is recruited continuously throughout the estrous cycle, with one follicle eventually being selected for ovulation (15). Each follicular wave generally consists of four parts, namely-recruitment, selection, deviation, and dominance (16, 17). In cattle, it was found that the first, second, and third follicular waves arise during the luteal phase, and this phase leads to the development of the dominant follicle (18). Moreover, in cattle, the number of dominant follicles is greater than in yak or buffalo; the size of the dominant follicle is >7 mm in diameter, and it continues to grow while the remaining smaller follicles undergo atresia (5). In a three-wave model, two- or three-wave follicular waves typically emerged around days 0, 9, and 16 of the estrous cycle (19).

Recent advances in our understanding of yak reproductive physiology have emphasized the importance of a detailed understanding of ovarian follicular dynamics and growth patterns for improving reproductive efficiency, implementing advanced reproductive technologies, and developing effective estrus synchronization strategies in this species. Therefore, this study aimed to characterize the number and pattern of follicular waves during the estrous cycle, evaluate follicular growth and regression dynamics using ultrasonography, and generate baseline data to support the development of fixed-time artificial insemination protocols in *Arunachali yak.*

## 2 Materials and methods

### 2.1 Experimental animals

The study was conducted on *Arunachali yak* maintained at the experimental animal farm of the ICAR-National Research Centre on Yak, Dirang, located in the West Kameng District of Arunachal Pradesh, India. A total of thirty-six (*n* = 36) apparently healthy cyclic female yaks of second to fourth parity and free from any post-parturient complications were selected for the study. The sample size used is comparable to that in previous studies investigating ovarian follicular dynamics in bovine species using ultrasonography. The animals were maintained under an intensive management system with standard feeding and the husbandry practices followed at the institute.

### 2.2 Management profile

The selected animals were housed separately under an intensive management system in a well-ventilated shed. They were maintained under a uniform dietary and management regimen consisting of green grasses, tree leaves, and a balanced concentrate mixture composed of maize grain, wheat bran, mustard oil cake, groundnut cake, mineral mixture, and common salt. The concentrate mixture was given at a rate of 2–2.5 kg per day in two divided portions: one in the morning (7:00 a.m.) and the other in the afternoon (4:00 p.m.). In addition, the animals were provided with paddy straw (5–6 kg/day) and green grasses (1–2 kg/day).

### 2.3 Experimental design

The thirty-six females yak were randomly allocated into two groups, namely, Group I and Group II, each consisting of 18 animals. Animals in Group I were maintained as the control without any hormonal treatment, whereas animals in Group II were subjected to a modified Ovsynch protocol specially designed for yak (Figure 1). On the day of the synchronization (Day 0), each animal of Group II received 500 μg (2 mL) of cloprostenol (Pragma, Intas Pharmaceutical Ltd., India), a prostaglandin (PGF2α) analog, administered intramuscularly. The modified Ovsynch protocol used in this study was adapted for yak based on previously reported synchronization strategies in bovines to improve ovulation synchronization and facilitate monitoring of follicular dynamics. After 48 h (Day 2) of the first cloprostenol treatment, animals received 10 μg of Buserelin acetate (Gynarich, Intas Pharmaceutical Ltd., India), a GnRH analog administered intramuscularly. Seven days after the first administration of Buserelin acetate (Day 9), a second dose of cloprostenol was administered with the same dose and route as the previous one. Two days after administration of the second dose of cloprostenol (Day 11), a second dose of Buserelin acetate was administered using the same dose and route as the first injection. Estrus in experimental animals was confirmed based on observable clinical signs of estrus, and all experimental animals were found to be cyclic. All the animals exhibiting estrus in both Group I and Group II were monitored for follicular and luteal dynamics for one estrous cycle (Day 0 to Day 21) using real-time transrectal ultrasonography obtained via an ultrasonic scanner (Model HS -2000, Honda Electronics Co., Ltd., Toyohashi, Aichi, Japan) equipped with a linear rectal probe (Model HLV – 475 M: 5–7.5 MHz) (Figure 2). All ultrasonographic examinations were performed by the same experienced operator to maintain consistency of measurements throughout the study. The diameter of the follicle and corpus luteum was measured using the electronic calipers integrated in the ultrasound system and recorded in millimeters.

**Figure 1:**
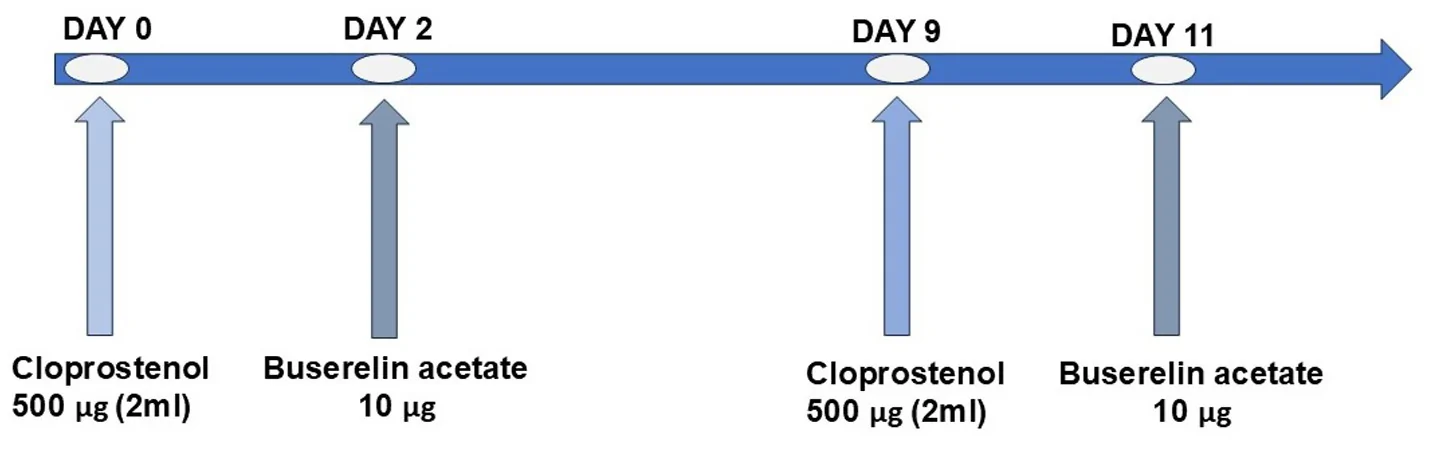
Modified Ovsynch protocol for group II *Arunachali Yak*.

**Figure 2:**
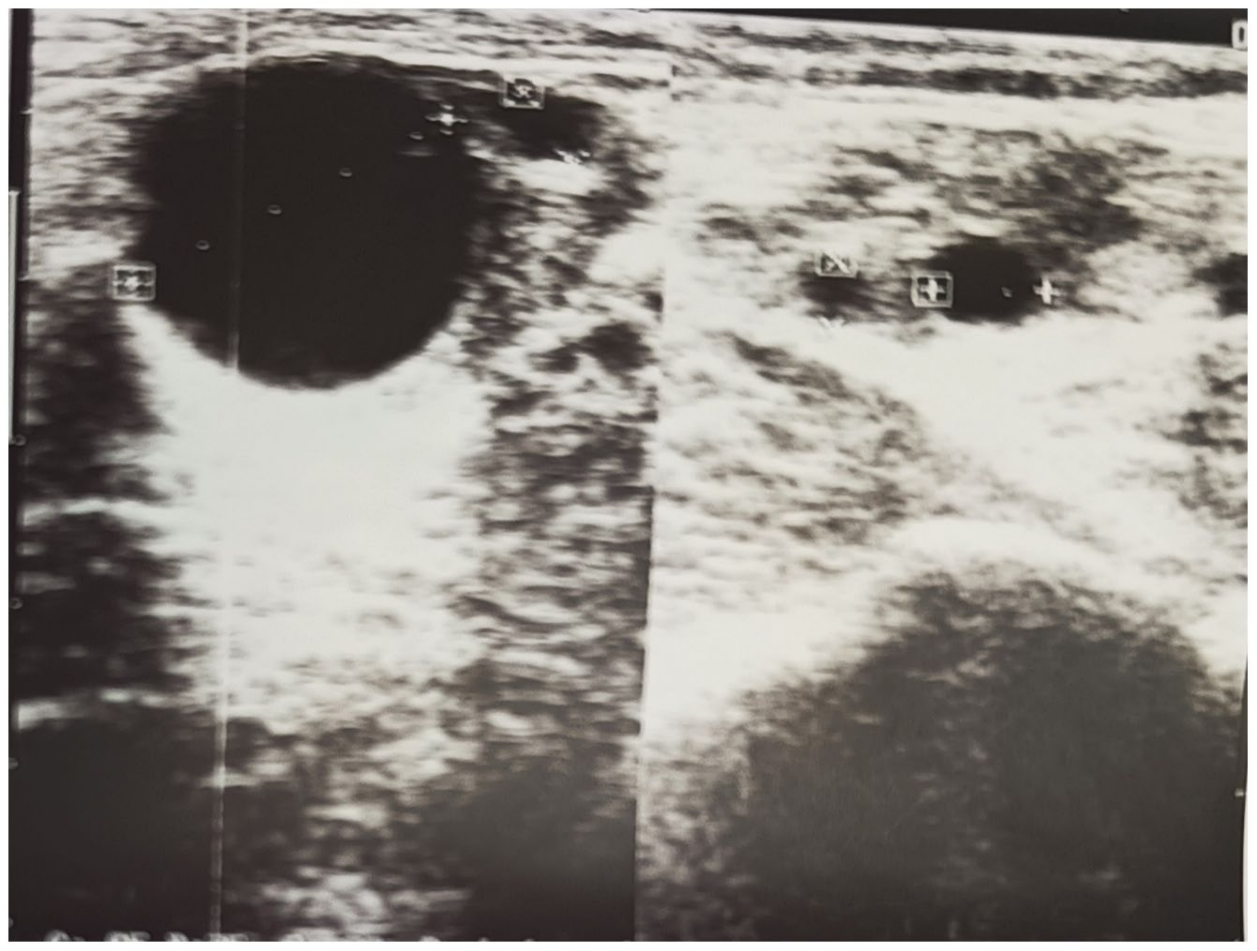
Transrectal ultrasonography in yak.

Ultrasonographic examinations were performed daily throughout the estrous cycle to monitor follicular growth, ovulation, and corpus luteum development.

### 2.4 Ultrasonographic observations of ovarian structures

Ovarian follicle evaluation was conducted from the day of estrus (Day 0) to Day 21 in both groups using real-time transrectal ultrasonography obtained via an ultrasonic scanner. Follicular growth was evaluated through ultrasonographic examination following the end of the synchronization protocol, from the day of estrus (Day 0) to Day 21.

### 2.5 Follicular dynamics

The length of the estrous cycle was calculated as the interval between two consecutive estrus events, considering the follicular wave pattern (20). The day of follicular wave emergence was defined as the day when one or more follicles ≥ 1.5 mm in diameter were first detected on the ovary by transrectal ultrasonography (20).

The duration of a follicular wave (wave length) was calculated as the time interval from the day of emergence (when a follicle or cohort of follicles first becomes detectable) to the day of atresia (disappearance or regression) of the dominant follicle (21). The number of recruited follicles was calculated as the total number of follicles ≥ 1.5 mm in diameter observed during a single follicular wave.

The life span of the dominant follicle (DF) was defined from the stage of follicular deviation. The life span of the anovulatory dominant follicle was calculated as the interval between the day of follicular deviation and the day of follicular atresia, while the life span of the ovulatory dominant follicle was calculated as the interval between the day of follicular deviation and the last day the follicle was observed before ovulation.

The dominant follicle (DF) was considered to be the largest follicle with a diameter >7.5 mm and was at least 2 mm larger than other follicles. The maximum diameter of the dominant ovulatory follicle and dominant anovulatory follicle was determined from daily ultrasonographic observations within the estrous cycle (22). The day of the estrous cycle on which the dominant follicle attained its maximum follicular diameter was also recorded.

### 2.6 Luteal dynamics

The life span of the corpus luteum (CL) was calculated as the interval between the day of CL regression and the day of the first appearance of the granular echogenic structure at the place of the DF after ovulation (23). Follicles that develop in the presence of a corpus luteum (CL) are dominant follicles from luteal-phase follicular waves, but they fail to ovulate and eventually regress. The maximum diameter of the corpus luteum was estimated as the mean of two perpendicular diameters (length and width) obtained using the built-in digital calipers of the ultrasonography scanner (24). The growing phase of the corpus luteum was defined as the number of days required for the CL to grow after ovulation to achieve its maximum diameter (25), while the regression phase of the corpus luteum was calculated as the number of days required for complete regression of the CL after reaching its maximum diameter (25). The ovulation rate in natural yak estrus was 100%, whereas, in induced estrus, it was 88.89%.

### 2.7 Endocrine profiling

Blood samples were collected daily from day of oestrus up to day 30 (early-, mid-, and late-luteal phases, as well as peri-estrus) to measure systemic hormone concentrations. Plasma concentrations of progesterone (P4), estradiol (E2), insulin-like growth factor 1 (IGF-1), and cortisol were quantified using validated immunoassay techniques following the manufacturer’s instructions. Intra- and inter-assay coefficients of variation (CV) were maintained below 10 and 12%, respectively, for all hormone assays.

### 2.8 Statistical analysis

The statistical analysis was conducted following the standard procedures described by Snedecor and Cochran (26). Descriptive statistics were used to calculate the mean, standard deviation, and standard error of the mean (SEM) for the variables studied. Differences between the natural estrus and induced estrus groups were evaluated using an independent-samples t-test assuming unequal variances. Each variable was assessed separately, as they represent distinct biological parameters; therefore, no adjustment for multiple comparisons was applied. A *p*-value of less than 0.05 was considered to indicate statistical significance. Post-hoc statistical power calculations were performed based on observed variance in estrous cycle length and luteal dynamics. The sample size of n equal to 18 per group (*N* = 36 total natural estrous vs. induced estrous) provided adequate statistical power (≥80%) at α = 0.05 to detect primary outcomes, including estrous cycle length and corpus luteum (CL) diameter. Daily ultrasonographic monitoring of cohort sizes of this scale is consistent with established high-altitude bovine reproductive literature.

## 3 Results

All animals included in the study (*n* = 36), comprising 18 animals in the natural estrus group (Group I) and 18 animals in the induced estrus group (Group II), exhibited estrus and completed one full estrous cycle under observation. Data from all animals were included in the analysis.

For standardization of observations, the day of estrous was considered to be Day 0 for both groups. Ovarian structures were monitored daily using transrectal ultrasonography from Day 0 until the completion of one full estrous cycle. All follicular and luteal parameters were recorded during this observation period.

### 3.1 Follicular dynamics

The overall mean estrous cycle length in natural estrus (Group I, *n* = 18) and induced estrus (Group II, *n* = 18) *Arunachali* female yak was found to be 20.00 ± 0.70 and 22.5 ± 0.35 days, respectively (Table 1). The overall estrous cycle length was significantly (*p* < 0.05) longer in the induced group compared to the natural group.

**Table 1:** Estrous cycle length (mean ± SEM).

Parameter	Natural (*n* = 18)	Induced (*n* = 18)	*p*-value
Length of estrous cycle (days)	20.00^a^ ± 0.70	22.50^b^ ± 0.35	0.02

^a,b^Means bearing different superscripts in a row differ significantly (*p* < 0.05).

The frequency of the occurrence of different follicular wave patterns is presented in Table 2. In the natural estrus group (Group I, *n* = 18), the two-wave pattern was most common (66.67%; *n* = 12), followed by the three-wave pattern (33.33%; *n* = 6) (Figures 3, 4). In contrast, in the induced estrus group (Group II, *n* = 18), the three-wave pattern was predominant (77.78%; *n* = 14), followed by the four-wave pattern (22.22%; *n* = 4).

**Table 2:** Frequency of occurrence (%) of different wave patterns of the estrous cycle in natural estrus and induced estrus yak cows.

Parameter	Natural		Induced	
	Two waves	Three waves	Three waves	Four waves
Length of estrous cycle in different wave patterns	19.60 ± 0.28	22.50 ± 1.40	22.33 ± 0.34	24.00 ± 0.41
Frequency of occurrence of different wave patterns of the estrous cycle in female *Arunachali Yak*	66.67	33.33	77.78	22.22

**Figure 3:**
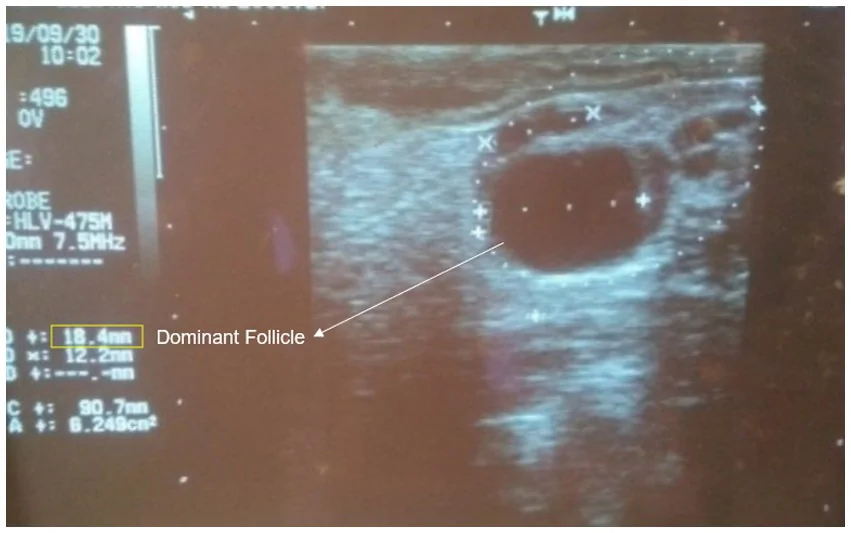
Dominant follicle on the day of heat (Day 0).

**Figure 4:**
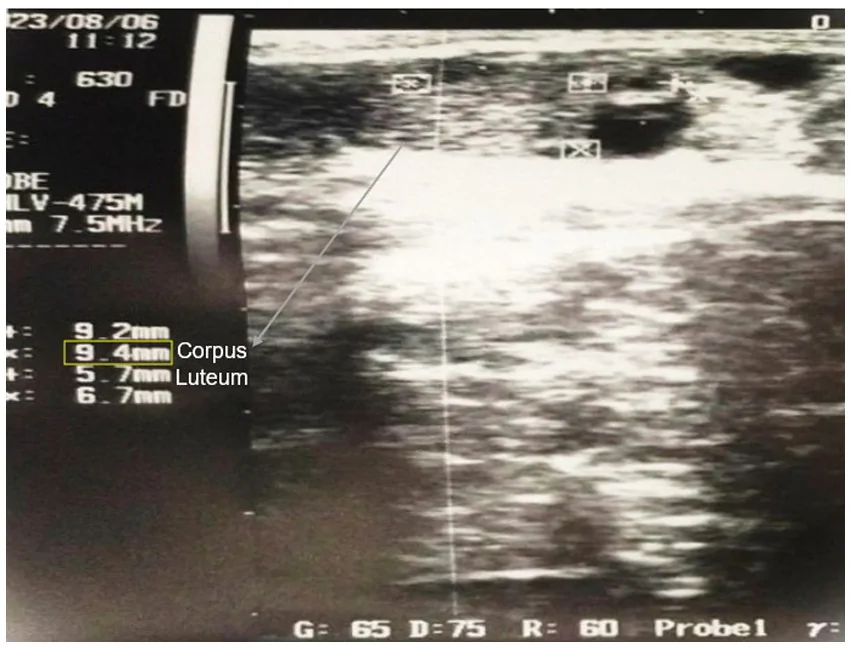
Corpus luteum at Day 4.

The mean day of emergence of the first (1.67 ± 0.12 vs. 1.75 ± 0.15 days), second (9.50 ± 0.15 vs. 8.50 ± 0.23 days), and third (16.00 ± 0.57 vs. 14.34 ± 0.22 days) follicular waves in Group I (natural) and Group II (induced) did not vary significantly between the groups (Table 3). In the induced estrus group (*n* = 4), the mean day of emergence of the fourth follicular wave was found to be Day 19.00 ± 0.40 of the estrous cycle (Table 3).

**Table 3:** Day at emergence of follicular wave.

Parameter	Natural (*n* = 18)	Induced (*n* = 18)	*p*-value
1st follicular wave	1.67 ± 0.12	1.75 ± 0.15	0.40
2nd follicular wave	9.50 ± 0.15	8.50 ± 0.23	0.19
3rd follicular wave	16.00 ± 0.57	14.34 ± 0.22	0.15
4th follicular wave		19.00 ± 0.40	

The pattern of follicular wave length showed a gradual decrease from the first to the third or fourth waves in both groups, with a shorter life span for the last follicular wave. The length of the first follicular wave was longer in both Group I (11.16 ± 0.21 days) and Group II (13.50 ± 0.12 days), and the length gradually decreased in the second (9.80 ± 0.17 vs. 9.12 ± 0.14 days) and third waves (7.00 ± 0.37 vs. 7.87 ± 0.07) in Group I and Group II, respectively (Table 2). The fourth follicular wave, observed only in Group II yak, was found to be shorter (6.00 ± 0.32 days) than the previous three waves (Table 4). No significant variation was recorded in the length of the follicular waves between groups. The number of recruited follicles ranged from 3 to 5 in the entire follicular wave pattern in both groups. (Table 5).

**Table 4:** Length of follicular wave (days).

Parameter	Natural (*n* = 18)	Induced (*n* = 18)	*p*-value
1st follicular wave	11.16 ± 0.21	13.50 ± 0.12	0.70
2nd follicular wave	9.80 ± 0.17	9.12 ± 0.14	0.26
3rd follicular wave	7.00 ± 0.37	7.87 ± 0.07	0.25
4th follicular wave		6.00 ± 0.32	

**Table 5:** Number of recruited follicles.

Parameter	Natural (*n* = 18)	Induced (*n* = 18)	*p*-value
1st follicular wave	4.50 ± 0.12	4.12 ± 0.16	0.07
2nd follicular wave	3.80 ± 0.14	4.38 ± 0.12	0.26
3rd follicular wave	4.40 ± 0.21	4.50 ± 0.12	0.25
4th follicular wave		3.0 ± 0.41	

There was a decreasing pattern in the life span of the dominant follicle from the first follicular wave to the third or fourth follicular wave in both groups. The life span of the dominant follicle from the stage of deviation to atresia in the first follicular wave differed significantly (*p* < 0.05) between the natural (7.53 ± 0.33 days) and induced (10.88 ± 0.41 days) estrus groups. In the second and third follicular waves, no significant difference was observed. The life span of dominant ovulatory follicles (third and fourth follicular waves) was numerically lower compared to that of dominant anovulatory follicles in Group I (4.50 ± 0.25 days) and Group II (4.00 ± 0.91 days) (Table 6).

**Table 6:** Life span of dominant follicle (DF) in days from stage of deviation.

Parameter	Natural (*n* = 18)	Induced (*n* = 18)	*p*-value
1st follicular wave	7.53^a^ ± 0.33	10.88^b^ ± 0.41	<0.05
2nd follicular wave	5.21 ± 0.25	5.75 ± 0.35	0.24
3rd follicular wave	4.50 ± 0.25	4.75 ± 0.27	0.37
4th follicular wave		4.00 ± 0.91	

^a,b^Means bearing different superscripts in a row differ significantly (*p* < 0.05).

The maximum diameter of dominant follicles was 12.46 ± 0.24 mm and 12.97 ± 0.23 mm in Group I and Group II, respectively, with no significant difference between groups (Table 7).

**Table 7:** Maximum diameter of dominant follicle (mm).

Parameter	Natural (*n* = 18)	Induced (*n* = 18)	*p*-value
1st follicular wave	12.98 ± 0.09	13.20 ± 0.15	0.07
2nd follicular wave	11.45 ± 0.11	10.93 ± 0.16	0.26
3rd follicular wave	13.25 ± 0.35	12.20 ± 0.34	0.25
4th follicular wave	–	12.60 ± 0.21	–
Maximum diameter of ovulatory follicles (mm)	12.46 ± 0.24	12.97 ± 0.23	0.25
Maximum diameter of anovulatory dominant follicles (mm)	12.28 ± 0.18	11.50 ± 0.17	0.15

The maximum size of the anovulatory dominant follicles was 12.28 ± 0.18 mm and 11.50 ± 0.17 mm in Group I and Group II, respectively, with no significant difference (*p* > 0.05). No significant variation was observed in the day at the maximum diameter of the dominant follicle or the growth rate of the dominant follicle between the groups (Table 7).

The diameter of the dominant follicles during the first, second and third follicular waves was 9.00 ± 0.18 mm vs. 9.38 ± 0.14 mm, 17.83 ± 0.22 mm vs. 16.00 ± 0.17 mm, and 22.00 ± 0.86 mm vs. 20.00 ± 0.18 mm in the Natural and Induced groups, respectively, with no significance difference between groups (Table 8).

**Table 8:** Day at maximum diameter of follicle.

Parameter	Natural (*n* = 18)	Induced (*n* = 18)	*p*-value
1st follicular wave	9.00 ± 0.18	9.38 ± 0.14	0.33
2nd follicular wave	17.83 ± 0.22	16.00 ± 0.17	0.07
3rd follicular wave	22.00 ± 0.86	20.00 ± 0.18	0.27
4th follicular wave		24.00 ± 0.41	

No significant differences were observed in the growth rate of dominant follicles between the Natural and Induced groups during the first (0.76 ± 0.02 vs. 0.92 ± 0.04 mm/day), second (0.77 ± 0.02 vs. 0.78 ± 0.03 mm/day), and third (1.35 ± 0.12 vs. 0.91 ± 0.03 mm/day) follicular waves. Data for forth follicular wave was available only in the induced group (1.17 ± 0.12), precluding comparison (Table 9).

**Table 9:** Growth rate of dominant follicle (mm/day).

Parameter	Natural (*n* = 18)	Induced (*n* = 18)	*p*-value
1st follicular wave	0.76 ± 0.02	0.92 ± 0.04	0.13
2nd follicular wave	0.77 ± 0.02	0.78 ± 0.03	0.46
3rd follicular wave	1.35 ± 0.12	0.91 ± 0.03	0.10
4th follicular wave		1.17 ± 0.12	

### 3.2 Subordinate follicle

There was no significant difference (*p* > 0.05) in the maximum diameter of the subordinate follicle between the first and second follicular wave in the natural estrus (Group I) and induced estrus (Group II) female yak. In contrast, a significant difference (*p* < 0.05) in the maximum diameter of the subordinate follicles were observed in the third follicular wave between Group I (6.60 ± 0.09 mm) and Group II (8.14 ± 0.09 mm) (Table 10).

**Table 10:** The maximum diameter of the subordinate follicle (mm).

Parameter	Natural (*n* = 18)	Induced (*n* = 18)	*p*-value
1st follicular wave	6.00 ± 0.19	6.50 ± 0.12	0.28
2nd follicular wave	13.83 ± 0.20	13.00 ± 0.14	0.26
3rd follicular wave	19.00 ± 0.73	18.63 ± 0.21	0.15
4th follicular wave		23.00 ± 0.41	

The day of maximum diameter of subordinate follicle during the first, second, and third follicular waves were 6.00 ± 0.19 vs. 6.50 ± 0.12 days, 13.83 ± 0.20 vs. 13.00 ± 0.14 days, 19.00 ± 0.73 vs. 18.63 ± 0.21 days in Natural and induced groups, respectively, with no significant difference between groups (Table 11).

**Table 11:** Day of maximum diameter of subordinate follicle.

Parameter	Natural (*n* = 18)	Induced (*n* = 18)	*p*-value
1st follicular wave	7.20 ± 0.07	8.00 ± 0.06	0.15
2nd follicular wave	6.73 ± 0.03	6.88 ± 0.05	0.38
3rd follicular wave	6.60a ± 0.09	8.14 b ± 0.09	0.03
4th follicular wave		7.50 ± 0.11	

In terms of the growth rate of the subordinate follicle, a significant (*p* < 0.05) difference was observed in the first follicular wave between Group I (0.40 ± 0.01 mm/day) and Group II (0.83 ± 0.04 mm/day). However, no significant variation was observed in the growth rate of the subordinate follicle in the second and third follicular wave between the groups (Table 12).

**Table 12:** Growth rate of subordinate follicle.

Parameter	Natural (*n* = 18)	Induced (*n* = 18)	*p*-value
1st follicular wave	0.40^a^ ± 0.01	0.83^b^ ± 0.04	<0.05
2nd follicular wave	0.68 ± 0.02	0.54 ± 0.05	0.21
3rd follicular wave	1.30 ± 0.08	0.96 ± 0.01	0.13
4th follicular wave		1.19 ± 0.18	

^a,b^Means bearing different superscripts in a row differ significantly (*p* < 0.05).

No significant differences were observed in the life span of the subordinate follicles from the stage of deviation between the Natural and Induced groups during the first (3.83 ± 0.07 vs. 3.88 ± 0.08 days), second (3.67 ± 0.06 vs. 3.81 ± 0.06 days), and third (2.50 ± 0.18 vs. 3.75 ± 0.09 days) follicular waves (Table 13).

**Table 13:** Life span of different subordinate follicles from the stage of deviation (days).

Parameter	Natural (*n* = 18)	Induced (*n* = 18)	*p*-value
1st follicular wave	3.83 ± 0.07	3.88 ± 0.08	0.47
2nd follicular wave	3.67 ± 0.06	3.81 ± 0.06	0.34
3rd follicular wave	2.50 ± 0.18	3.75 ± 0.09	0.09
4th follicular wave		3.00 ± 0.41	

### 3.3 Luteal dynamics

The life span of the corpus luteum in Group I and Group II was 18.25 ± 0.19 days and 19.25 ± 0.18 days, respectively, with no significant variation between the groups. The maximum diameter of the corpus luteum was significantly (*p* < 0.05) larger in Group II (21.16 ± 0.11 mm) compared to Group I (18.23 ± 0.12 mm) (Figure 5). The day at the maximum diameter of the corpus luteum in Group I and Group II was 10.13 ± 0.16 and 11.00 ± 0.19 days, respectively, with no significant variation between the groups. The growing phase of the corpus luteum was 11.63 ± 0.16 and 12.63 ± 0.14 days in Group I and Group II, respectively, with no significant variation (Table 14).

**Figure 5:**
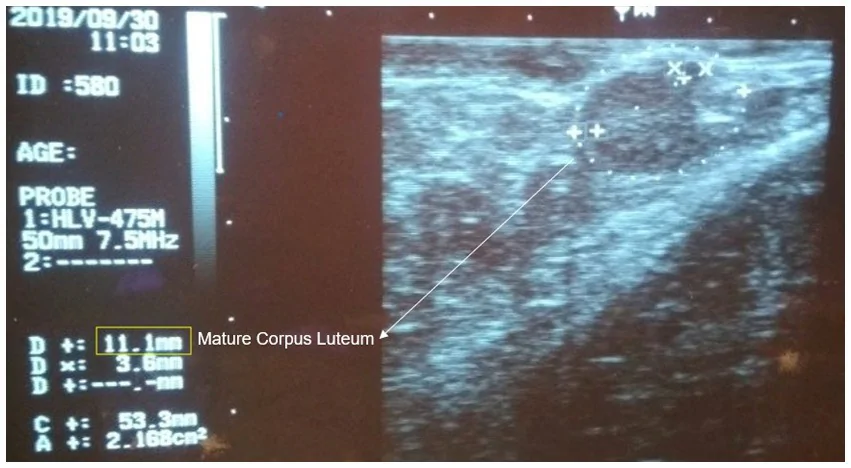
Mature corpus luteum with maximum diameter at Day 11.

**Table 14:** Details of the life span, maximum diameter, day at maximum diameter, and growing and regressing phases of the corpus luteum in natural and induced estrus in female yak.

Parameters	Natural (*n* = 18)	Induced (*n* = 18)	*p*-value
Life span of corpus luteum (days)	18.25 ± 0.19	19.25 ± 0.18	0.20
Maximum diameter of corpus luteum (mm)	18.23^a^ ± 0.12	21.16^b^ ± 0.11	<0.05
Day at maximum diameter of corpus luteum	10.13 ± 0.16	11.00 ± 0.19	0.15
Growing phase of corpus luteum (days)	11.63 ± 0.16	12.63 ± 0.14	0.21
Regression phase of corpus luteum (days)	6.63 ± 0.16	7.63 ± 0.21	<0.05

^a,b^Means bearing different superscripts in a row differ significantly (*p* < 0.05).

The regression phase of the corpus luteum was significantly (*p* < 0.05) longer in Group II (7.63 ± 0.21 days) compared to Group I (6.63 ± 0.16 days) (Figure 6).

**Figure 6:**
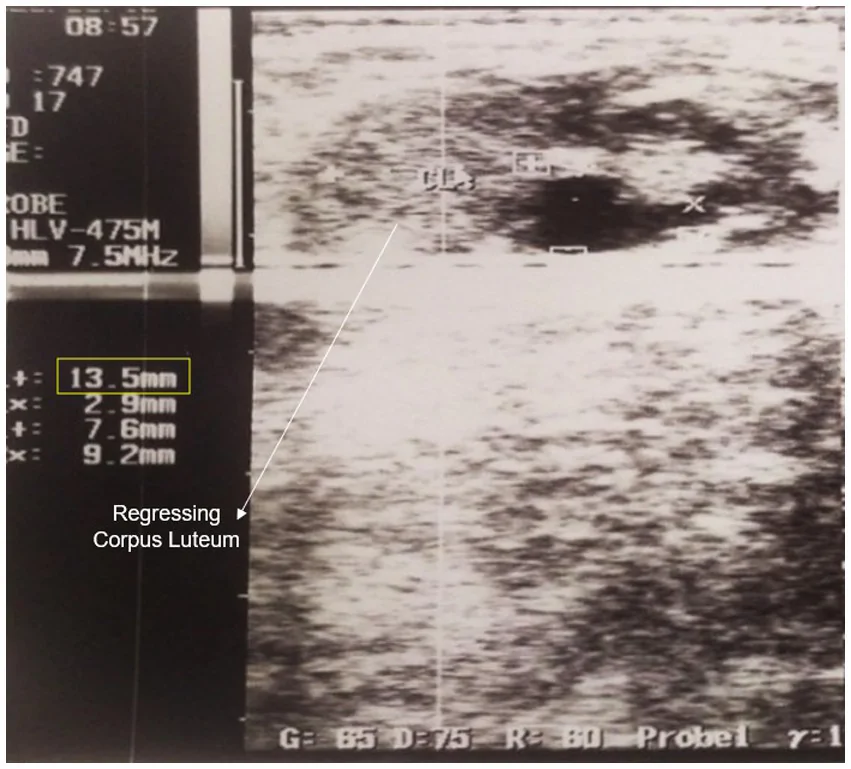
Regressing corpus luteum at Day 17.

### 3.4 Endocrine profiles across the estrous cycle

Endocrine profiling revealed significant variations between the natural and induced estrus groups across cycle phases. Induced animals exhibited significantly higher progesterone (P4) concentrations during the first (2.21 ± 0.17), second (4.68 ± 0.14), and third (5.75 ± 0.13) ng/ml peak phases compared to natural control animals (1.67 ± 0.15, 3.85 ± 0.13, 4.96 ± 0.18 ng/mL), respectively, (*p* < 0.05) (Figure 7). Pre-ovulatory estradiol (E2) peaks were significantly higher in the induced group (29.20 ± 3.19) pg./ml (*p* < 0.05) (Figure 8). Additionally, insulin-like growth factor 1 (IGF-1) concentrations were significantly elevated during the mid- 98.76 ± 2.84 ng/mL and late-luteal 124.63 ± 2.97 ng/mL phases in induced animals (*p* < 0.05) (Figure 9). Plasma cortisol levels were also significantly higher in the induced group during the early- (3.47 ± 0.25) and mid-luteal phases (3.14 ± 0.09) (*p* < 0.05) (Figure 10).

**Figure 7:**
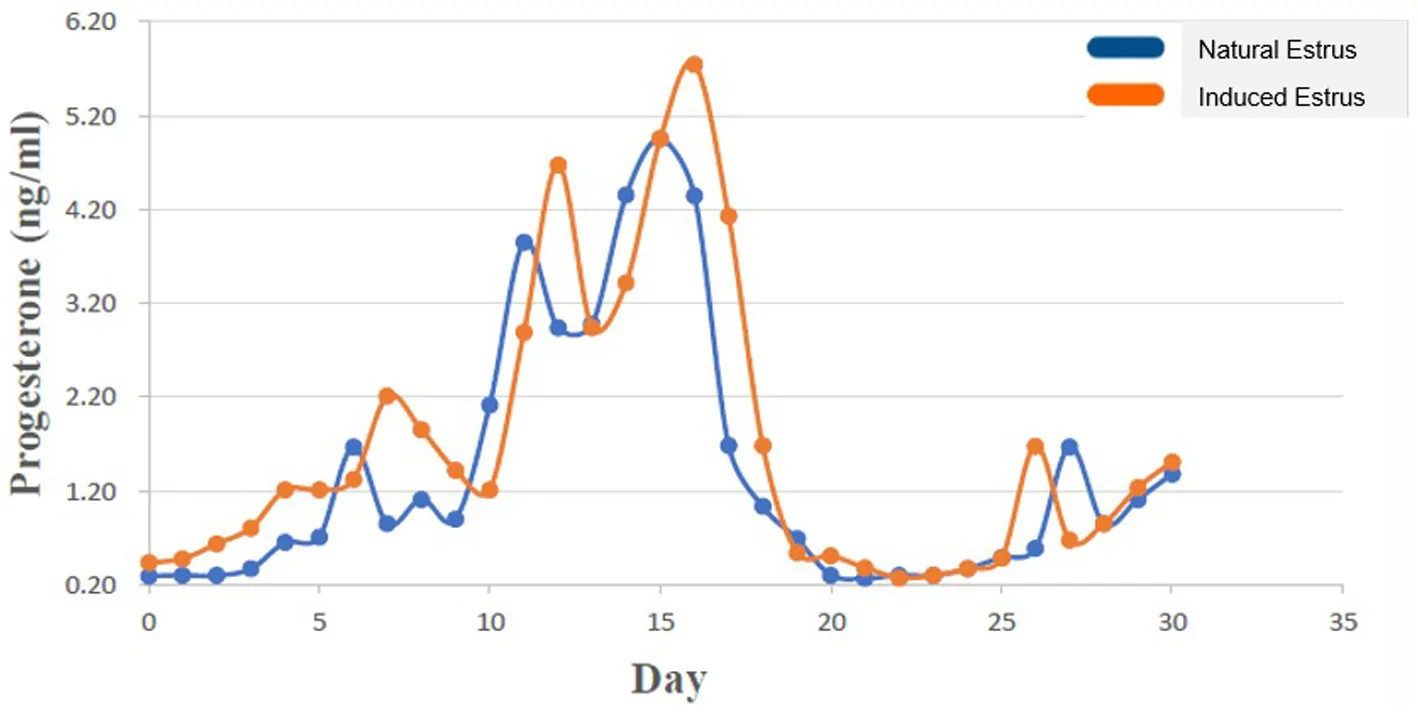
Variation in serum progesterone level in different phases of estrous cycle.

**Figure 8:**
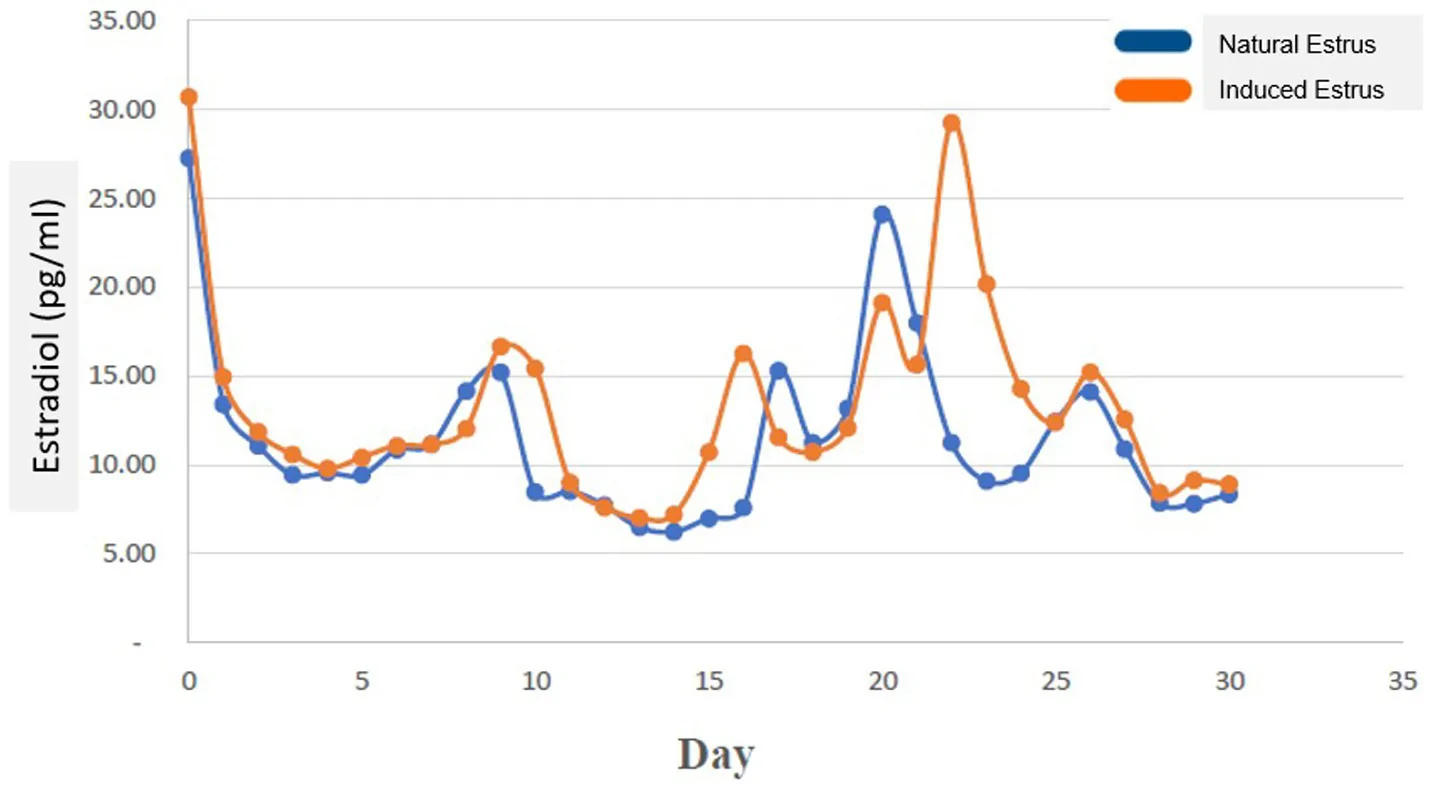
Variation in serum estradiol level in different phases of estrous cycle.

**Figure 9:**
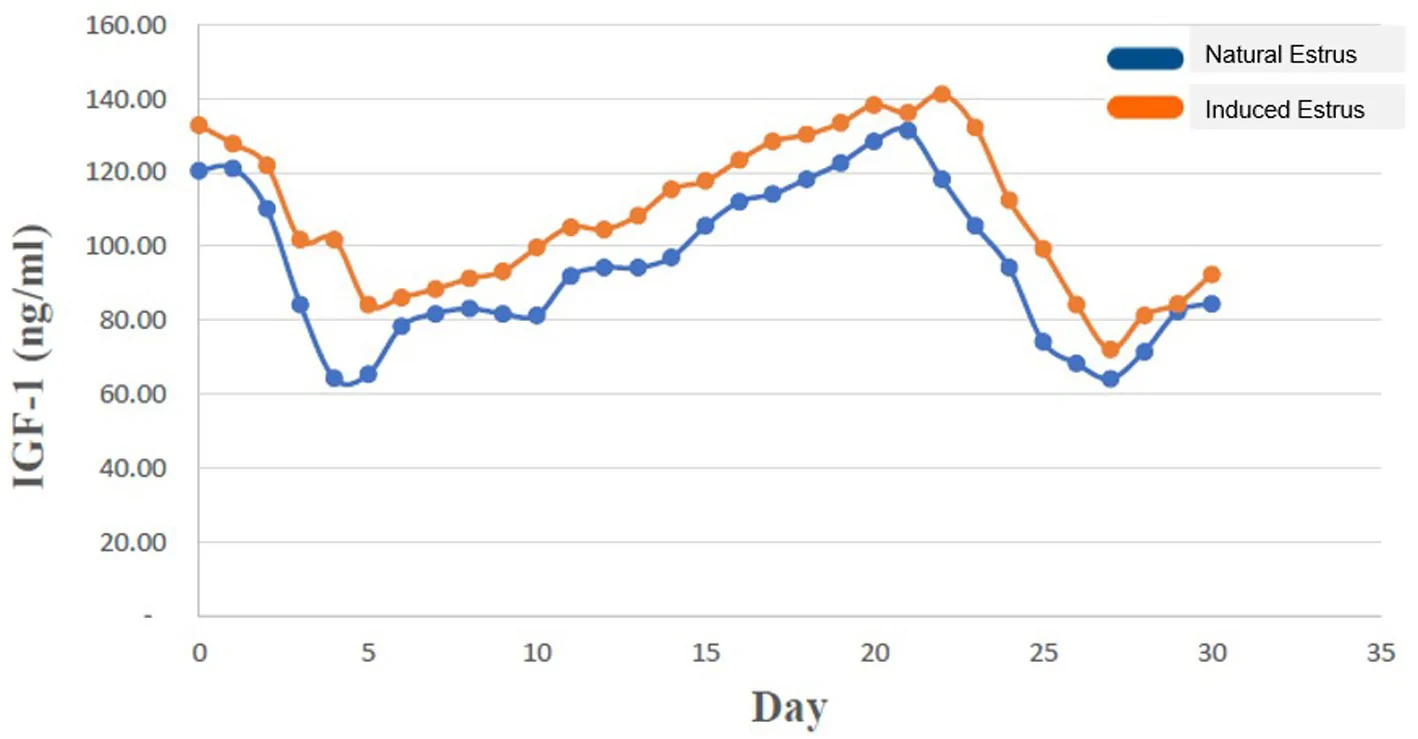
Variation in serum IGF-1 level in different phases of estrous cycle.

**Figure 10:**
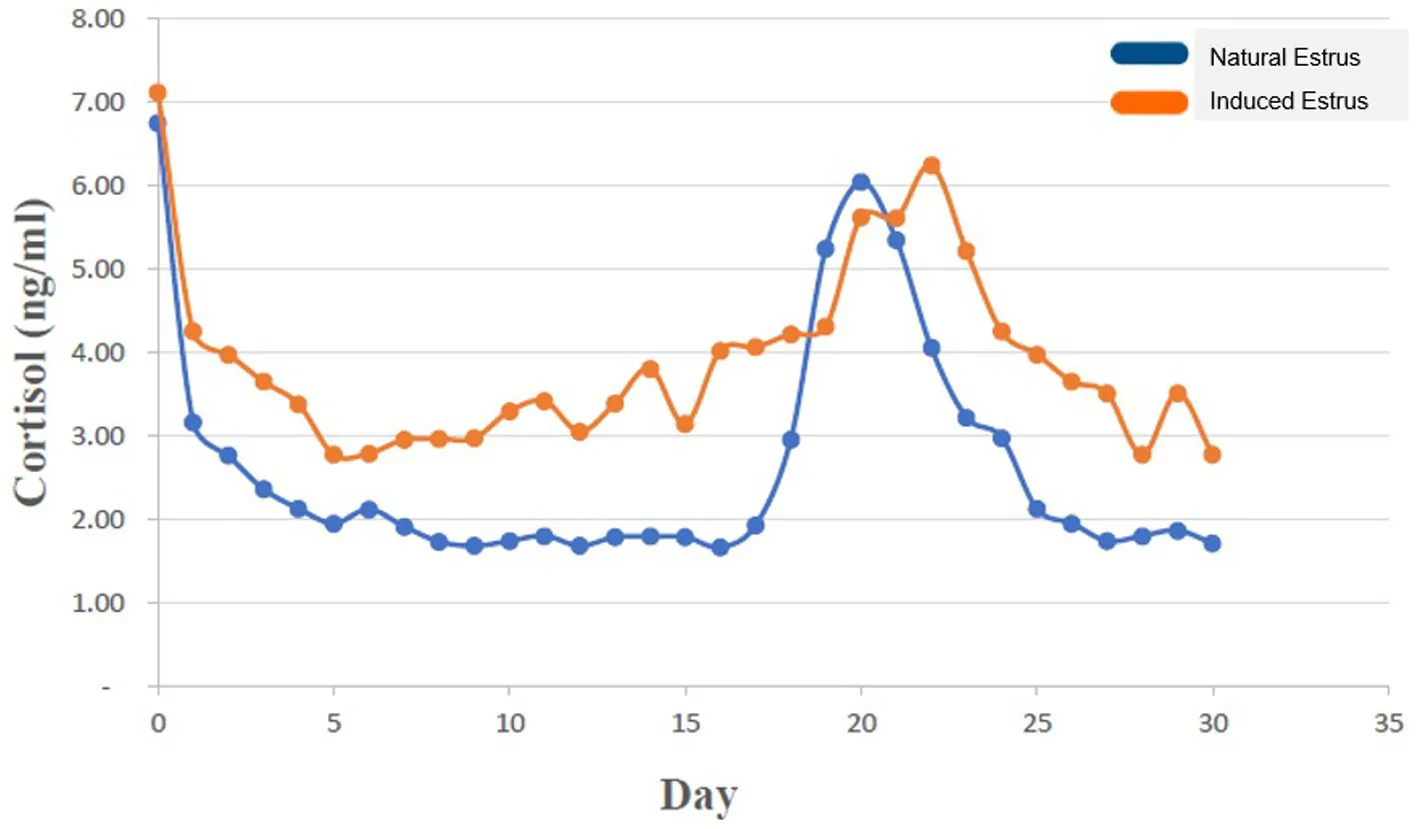
Variation in serum cortisol level in different phases of estrous cycle.

## 4 Discussion

This study was conducted to investigate and compare follicular and luteal dynamics during natural and hormonally induced estrous cycles in *Arunachali yak.* The findings revealed clear differences between the two groups, particularly in follicular wave patterns, estrous cycle length, and corpus luteum characteristics. Natural estrus was predominantly associated with a two-wave follicular pattern, whereas induced estrus showed a higher frequency of three- and four-wave patterns, along with a significantly longer estrous cycle and larger corpus luteum.

The higher frequency of two follicular waves observed in this study is in agreement with earlier reports on cattle and *Bos taurus* and Nellore cows (21, 24, 27–29). However, contrary to our findings, some studies reported a lower frequency of two follicular wave patterns compared with three- and four-wave patterns during the estrous cycle of cattle heifers (26, 30, 31). Mithun cows have also been reported to exhibit a higher frequency of three follicular wave patterns during induced estrus, along with a longer estrous cycle length in animals showing four follicular waves, which corroborates the findings of the present study (15). The longer estrous cycle length observed in animals exhibiting four follicular waves patterns may be attributed to a longer mean inter-ovulatory interval caused by delayed regression of the corpus luteum (CL), as luteal regression generally occurs after the emergence of the ovulatory wave (2, 15, 32).

Follicular recruitment is influenced by the available pool of primordial follicles as well as endocrine stimulation. The lower recruitment of follicles observed in yak may also be associated with species-specific reproductive adaptations, as yak are high-altitude bovines with unique endocrine regulation and metabolic adaptations that can influence ovarian activity and follicular development (33, 34). These physiological adaptations may contribute to differences in follicular dynamics between yak and other bovine species. Similar findings regarding the life span of the dominant follicle have been reported in cattle heifers (2, 35, 36), although some studies in cattle have documented a longer life span of the dominant follicle (26, 37–39).

The higher occurrence of multiple follicular waves in the induced estrus group may be attributed to the hormonal interventions used in the synchronization protocol. Administration of GnRH and prostaglandin likely altered the endocrine environment, leading to repeated recruitment of follicular waves and delayed section of the ovulatory follicle. This extended follicular activity may explain the longer estrous cycle observed in hormonally treated animals. In contrast, the predominance of a two-wave pattern in natural estrus suggests a more physiologically regulated follicular turnover under natural conditions. The prolonged estrous cycle observed in induced estrus animals could be associated with delayed luteal regression and the emergence of additional follicular waves before ovulation since luteolysis is closely linked with the timing of the ovulatory interval. This finding is important as it indicates that synchronization protocols may alter not only the timing of ovulation but also the overall cycle dynamics in yak.

The significantly larger corpus luteum observed in the induced estrus group is likely due to the effect of GnRH administration, which promotes ovulation and enhances luteinization of follicular cells. A larger corpus luteum may be associated with increased progesterone secretion, which plays a critical role in maintaining pregnancy. Additionally, the longer regression phase of the corpus luteum in induced estrus animals further support the observation of an extended luteal phase in these animals. The observed pattern of decreasing follicular wave length and dominant follicle life span across successive waves in both groups indicates a progressive decline in follicular dominance as the cycle advances. This may be due to changes in hormonal support, particularly fluctuation in FSH and LH, which regulate follicular growth and atresia. The significantly higher concentration of progesterone in induced estrous group may be due to the administration of the first GnRH injection in the modified synchronization protocol (PGF2α + GnRH+ PGF2α + GnRH), which may have stimulated ovulation and luteinization of growing follicles, followed by increase corpus luteum differentiation and function, leading to increased progesterone secretion (40, 41). The present findings are in consistent with the findings where they reported similar progesterone concentration *viz.* 0.84 ± 0.32, 1.63 ± 0.07 and 3.67 ± 0.99 ng/mL in the early, mid and late luteal phase, respectively, during oestrous cycle in female yak (42). Similar increased level of progesterone reported in Mithun, Thai native heifer (37, 43).

The serum estradiol concentration was significantly higher in induced female yaks at subsequent estrous compared to naturally cycling yaks. This could be related to the higher number of follicular waves in the induced group, suggesting the presence of relatively more growing or dominant follicles that contribute to circulating estradiol concentration. Similar estradiol concentration at subsequent natural estrous reported in Mithun cow (43). Estradiol concentration was significantly higher at the third peak in the induced group, but not significantly different between groups at the first and second peaks. The higher concentration at the third peak may be related to the effect of GnRH administration during the preceding estrus that may have promoted follicular development and estrogen synthesis. A lower first-peak estradiol concentration reported in llamas during the estrous cycle with the present findings (44).

Higher circulating IGF-1 during the mid- and late-luteal phases further supports increased metabolic and gonadotropic drive in induced cycles. Elevated cortisol concentrations during the early- to mid-luteal phases highlight potential physiological or handling-induced stress responses associated with synchronization protocols.

From a practical perspective, these findings have important implications for reproductive management in yak. Understanding the differences in follicular dynamics between natural and induced estrus can help refine estrus synchronization protocols and improve the timing of artificial insemination. The identification of predominant follicular wave patterns and ovulatory timing is particularly useful for developing fixed-time artificial insemination (FTAI) strategies, which can overcome the challenges of poor estrus expression and detection in yak. Ultimately, such improvements can enhance reproductive efficiency, genetic progress, and the livelihood security of high-altitude farming communities.

## 5 Conclusion

The present study provides insights into ovarian follicular dynamics, corpus luteum development and endocrine profiles during natural and hormonally induced estrous cycles in *Arunachali yak*. The findings indicate that hormonal induction of estrus can influence follicular wave patterns, luteal characteristics and endocrine profile compared with natural estrous cycles. Natural estrus was predominantly characterized by a two-wave follicular pattern, whereas hormonally induced cycles showed a greater frequency of three- and four-wave patterns along with a prolonged estrous cycle, larger corpus luteum and extended luteal regression. These alterations may reflect changes in follicular recruitment, ovulatory timing and luteal function following hormonal synchronization. The predominance of two-wave cycles under natural conditions and the increased occurrence of multiple follicular waves in induced cycles indicate differences in ovarian dynamics between natural and hormonally synchronized cycles. The endocrine profile supported these ovarian changes with higher progesterone concentrations in induced cycles potentially reflected enhanced ovulation, luteinization and corpus luteum function following GnRH administration. Higher estradiol concentrations at subsequent estrus and during the third follicular peak were associated with increased follicular activity. Increased IGF-1 during the mid- and late-luteal phases may indicate enhanced metabolic support of ovarian function, whereas elevated cortisol during the early- to-mid luteal phase suggests an altered physiological or handling-associated stress response. As a whole, these findings demonstrate that hormonal induction modifies follicular wave dynamics, luteal development and the associated endocrine milieu compared with natural estrous cycles. These differences may be associated with the distinctive reproductive and metabolic adaptations of yak to high-altitude environments. The study contributes to a better understanding of reproductive physiology in *Arunachali yak* and provides a physiological basis for refining estrus synchronization and fixed-time artificial insemination (FTAI) protocols to improve reproductive efficiency and genetic dissemination in high-altitude yak production systems.

## Data Availability

The raw data supporting the conclusions of this article will be made available by the authors, without undue reservation.
